# A nomogram for predicting lateral lymph node metastasis in cN0 unifocal papillary thyroid microcarcinoma

**DOI:** 10.1186/s12885-023-11219-0

**Published:** 2023-08-01

**Authors:** Hui Huang, Siyuan Xu, Song Ni, Xiaolei Wang, Shaoyan Liu

**Affiliations:** 1 Department of Head and Neck Surgical Oncology, National Cancer Centre, National Clinical Research Centre for Cancer/Cancer Hospital, Chinese Academy of Medical Sciences and Peking Union Medical College, No. 17, Panjiayuan Nanli, Chaoyang District, Beijing, 100021 China; 2 Department of Otolaryngology Head and Neck Surgery, Beijing Tongren Hospital, Capital Medical University/Key Laboratory of Otolaryngology Head and Neck Surgery, Ministry of Education, Beijing Institute of Otolaryngology, Beijing, China

**Keywords:** Papillary thyroid microcarcinoma, Clinical negative lymph node, Lateral lymph node metastasis, Risk factors

## Abstract

**Background:**

Identifying risk factors for occult lateral lymph node metastasis (LLNM) in papillary thyroid microcarcinoma (PTMC) can provide valuable insights into the necessity of lateral neck dissection (LND). The objective of this study was to develop a nomogram for predicting the probability of LLNM in patients with cN0 unifocal PTMC.

**Methods:**

We conducted a retrospective analyzed a total of 4872 patients with cN0 unifocal PTMC who were treated at our center from January 2013 to June 2018. Logistic regression analysis was used to determine the risk factors for LLNM, and a nomogram was constructed based on these risk factors.

**Results:**

The rate of LLNM was 3.2%. Tumors located in the upper lobe(odds ratio [OR] = 2.56, 95% confidence interval [CI] 1.80–3.62; p < 0.001) and size greater than 7 mm (OR = 2.59, 95% CI 1.85–3.62; p < 0.001) had a significantly higher risk of LLNM compared to tumors in the lower or middle lobe and size less than or equal to 7 mm. Tumors with extrathyroidal extension (ETE) had a significantly higher risk of LLNM (OR = 1.41, 95% CI 1.01–1.99; p = 0.044). The presence of three or more central lymph node metastases (CLNMs) (OR = 5.84, 95% CI 3.83–8.93; p < 0.001) or one or two CLNMs (OR = 2.91, 95% CI 1.93–4.42; p < 0.001) also increased the risk of LLNM compared to having no CLNMs. A nomogram incorporating these risk factors was developed, and the receiver operating characteristic (ROC) curve demonstrated an area under the curve (AUC) of 0.777, indicating a high degree of predictive accuracy.

**Conclusion:**

Tumor location in the upper lobe, greater than 7 mm in size, ETE, and CLNMs, especially three or more, were independent risk factors for LLNM in cN0 unifocal PTMC. The nomogram based on these factors exhibited favorable predictive value and consistency.

## Background

Papillary thyroid microcarcinoma (PTMC), which refers to papillary thyroid carcinoma (PTC) with a largest diameter of ≤ 1 cm [1], contributes significantly to the increase in incidence of PTC [2]. Although PTMC patients generally have a good prognosis, with a 10-year disease-specific survival rate as high as 99% and a low recurrence rate of 5% or less at the site of surgery [3, 4], lymph node metastases (LNM) in the cervical region occur in approximately 3.1–64% of cases [5–7]. According to guidelines from the American Thyroid Association (ATA), prophylactic central neck dissection (pCND) is recommended for patients with clinically negative cervical lymph nodes (cN0) who have advanced primary tumors (cT3/4) or clinically involved lateral neck nodes (cN1b) [8]. Many studies have examined the risk factors for central LNM in cN0 PTMC [9]. For the lateral neck, the ATA recommends therapeutic lateral neck dissection (LND) for cN1b PTC [8]. The incidence of lateral LNM(LLNM) in PTMC has varied in previous studies, ranging from 3.7 to 44.5% [6, 10–12]. Most studies include both palpable and nonpalpable lymph nodes when analyzing the incidence of lateral LNM [6]. However, till now, no research has been conducted on the nomogram for LLNM in cN0 PTMC, particularly with a substantial sample size. Studies have demonstrated that LNM in PTMC, particularly in the lateral neck region, is strongly linked to tumor recurrence and disease-free survival [13, 14]. Identifying risk factors for LLNM can assist in determining the necessity of LND and other measures to reduce the risk of recurrence. The aim of this retrospective study is to determine clinicopathological risk factors for LLNM in cN0 unifocal PTMC and develop a practical nomogram for predicting the probability of LLNM, guiding therapeutic decision-making for surgeons.

## Materials and methods

### Patients

We retrospectively reviewed the medical records of 15,720 patients with papillary thyroid carcinoma (PTC) who underwent initial surgery at our center between January 2013 and June 2018. Among them, 9,929 patients had PTC no larger than 10 mm on pathology (PTMC), while 747 patients had clinically positive central or lateral cervical lymph nodes (cN1a/b). Among the 9,182 patients with cN0 PTMC, 685 did not receive prophylactic central neck dissection (pCND). We further reviewed the data of the 8,497 patients who received unilateral or bilateral pCND with or without lateral neck dissection (LND). A total of 376 patients with incomplete clinicopathological results were excluded, and 4,872 patients with unifocal PTMC diagnosed by postoperative pathology were finally included. Clinical lymph node (LN) status was determined according to the preoperative ultrasound (US) results. cN0 was diagnosed if no suspicious features, such as focal or diffuse hyperechogenicity, internal calcifications, cystic changes, or round shape, were observed on US [15].

Surgery included either lobectomy + isthmectomy or total thyroidectomy plus ipsilateral or bilateral pCND. The ipsilateral CND included prelaryngeal, pretracheal, and paratracheal lymph nodes. In cases where the preoperative US showed enlarged lateral lymph nodes, intraoperative frozen pathological examination was performed, and LND was conducted for patients with positive lymph nodes.

### Data collection

The collected data included sex, age, tumor size, tumor location, Hashimoto’s thyroiditis (HT), multifocality, bilaterality, extrathyroidal extension (ETE), lymphovascular invasion (LVI), pathological central lymph node (LN) status, and lateral LN status. Age was dichotomized according to the current stage standards at 55 years. Tumor size was recorded based on the largest tumor dimension, and the receiver operating characteristic (ROC) curve revealed a significant tumor size cutoff value of 7.5 mm (integrated area under the curve = 68.2%; *P* = 0.000, 95% CI = 0.640–0.724). Patients were divided into two groups based on tumor size (≤ 7 mm and > 7 mm). Diagnosis of HT, multifocality, bilaterality, ETE, and LVI was based on pathological results. For a unifocal tumor, the tumor location was recorded as upper, middle, or lower based on intraoperative findings.

Staging was performed according to the American Joint Committee on Cancer TNM Stage for Thyroid Cancer (8th Edition, 2017) [16]. The initial risk stratification was performed according to the 2015 American Thyroid Association (ATA) guidelines [8].

### Statistical analysis

We performed univariate and multivariate analyses using SPSS v27.0 (SPSS Inc., Chicago, IL, USA) software. Fisher’s chi-squared test was used to examine intergroup differences in categorical variables. Logistic regression tests were carried out to determine variables associated with LLNM. Statistically significant intergroup differences were defined by p-values < 0.05. We applied the R package “rms” version 6.3 to construct the nomogram, including risk-factor screening using logistic regression to predict LLNM in patients with cN0 unifocal PTMC. The length of the line corresponding to each factor on the nomogram reflected the contribution of each factor to LLNM. The risk score was calculated using the R package “nomogram Formula” version 1.2. We examined the predictive value of LLNM using the calibration curves. The data were analyzed using R software version 4.2.2. Receiver operating characteristic (ROC) curves were used to evaluate the predictive accuracy of the nomogram and determine the cutoff values. We calculated the thresholds with the highest sensitivity–specificity sum using the R package “ROCR” version 1.0–11 and plotted them on the ROC curve. We considered p < 0.05 to be statistically significant.

## Results

### Clinicopathological characteristics

The study population included 4872 patients, whose characteristics are summarized in Table 1. The average age was 43.46 ± 10.36 years (range: 13–77). The average tumor size was 6.3 ± 2.1 mm (range: 1–10). Among the 4872 patients, 3171 (65.1%) underwent ipsilateral lobectomy + isthmectomy, 818 (16.8%) underwent ipsilateral lobectomy + isthmectomy + contralateral partial thyroidectomy, and 883 (18.1%) underwent bilateral total thyroidectomy. A total of 4693 (96.3%) patients underwent ipsilateral pCND, while 179 (3.7%) underwent bilateral pCND. A total of 1815 (37.3%) had LNM in the ipsilateral central neck (CLNM). Twenty-nine (0.6%) patients had bilateral CLNM. The average number of positive LNs in the central neck was 2.50 ± 2.04 (range: 1–19). A total of 157 (3.2%) patients had lymph node metastasis in the lateral neck (LLNM). Among the 3027 patients with no CLNM, 47 (1.6%) patients had LLNM. The average number of positive LNs in the lateral neck was 2.45 ± 1.74 (range: 1–9). The LND included level II-V in 16 patients and level II-IV in 75 patients. Sixty-six patients with intraoperative frozen negative LN results but positive LN results diagnosed by postoperative paraffin pathology did not undergo LND. Overall, 3371 (69.2%) patients had tumors ≤ 7 mm, and 1501 patients (30.8%) had tumors > 7 mm. Of the 2124 (43.6%) patients with ETE, 2012 patients had microscopic ETE (micro-ETE), while 112 (2.3%) had macroscopic ETE (macro-ETE). The rates of LLNM in patients with macro-ETE and without macro-ETE were 3.6% (4/112) and 3.2% (153/4607), respectively, with no significant difference (OR = 1.12, 95% CI 0.34–2.70; p = 0.833).

**Table 1:** Baseline Clinicopathological Characteristics

Characteristic		N = 4872
Sex	Female	3,668 (75.3%)
	Male	1,204 (24.7%)
Age group	≥ 55 years	724 (14.9%)
	<55 years	4,148 (85.1%)
Tumor size	≤ 7 mm	3,371 (69.2%)
	>7 mm	1,501 (30.8%)
Tumor location	upper	1,143 (23.5%)
	middle	2,534 (52.0%)
	lower	1,195 (24.5%)
Hashimoto’s thyroiditis		1,093 (22.4%)
LVI		31 (0.6%)
ETE		2,124 (43.6%)
ENE		175 (3.6%)
pT stage	1a	4,760 (97.7%)
	3b	90 (1.8%)
	4a	22 (0.5%)
pN stage	0	2,988 (61.3%)
	1a	1,727 (35.4%)
	1b	157 (3.2%)
pTNM stage	I	4,676 (96.0%)
	II	192 (3.9%)
	III	4 (0.1%)
ATA risk	low	2,642 (54.2%)
	intermediate	2,117 (43.5%)
	high	112 (2.3%)

### Risk factors for LLNM in patients with cN0 unifocal PTMC

In the univariate analysis, LLNM was significantly associated with sex, tumor size, tumor location, ETE, and number of CLNMs (Table 2). The LLNM rates of patients with tumors in the lower, middle, and upper lobes were 2.5%, 2.7%, and 5.1%, respectively. Tumors in the upper lobe had a significantly higher risk of LLNM than tumors in the lower lobe (OR = 2.08, 95% CI 1.34–3.29; p = 0.001), but there was no difference in the LLNM rate between patients with tumors in the middle and lower lobes (OR = 1.09, 95% CI 0.71–1.70; p = 0.706).

In the multivariate analysis (Table 2), no differences were found between female and male patients (OR = 1.35, 95% CI 0.94–1.93; p = 0.102). Tumors in the upper lobe had a significantly higher risk of LLNM than tumors in the lower and middle lobes (OR = 2.56, 95% CI 1.80–3.62; p < 0.001). Tumor size > 7 mm was associated with a significantly higher risk of LLNM compared with tumor size ≤ 7 mm (OR = 2.59, 95% CI 1.85–3.62; p < 0.001). Patients with ETE had a higher risk of LLNM metastasis (OR = 1.41, 95% CI 1.01–1.99; p = 0.044). Having ≥ 3 CLNMs (OR = 5.84, 95% CI 3.83–8.93; p < 0.001) or 1–2 CLNMs (OR = 2.91, 95% CI 1.93–4.42; p < 0.001) significantly increased the risk of LLNM compared with those with no CLNMs.

**Table 2:** Univariate and Multivariate Analyses of Risk Factors using Logistic Regression Analysis

		LLNM		OR (95% CI, p value)	
		**no**	**yes**	**univariable**	**multivariable**
Sex	Female	3565 (97.2)	103 (2.8)	-	-
	Male	1150 (95.5)	54 (4.5)	1.63 (1.15–2.26, 0.005)	1.35 (0.94–1.93, 0.102)
Age group	≥ 55 years	705 (97.4)	19 (2.6)	-	-
	<55 years	4010 (96.7)	138 (3.3)	1.28 (0.81–2.14, 0.324)	0.97 (0.60–1.65, 0.903)
Tumor upper location	no	3630 (97.3)	99 (2.7)	-	-
	yes	1085 (94.9)	58 (5.1)	1.96 (1.40–2.72,0.001)	2.56 (1.80–3.62,0.001)
Tumor size	≤ 7 mm	3308 (98.1)	63 (1.9)	-	-
	>7 mm	1407 (93.7)	94 (6.3)	3.51 (2.54–4.87,0.001)	2.59 (1.85–3.65,0.001)
Hashimoto’s thyroiditis	no	3661 (96.9)	118 (3.1)	-	-
	yes	1054 (96.4)	39 (3.6)	1.15 (0.78–1.64, 0.463)	1.27 (0.85–1.87, 0.224)
ETE	no	2684 (97.7)	64 (2.3)	-	-
	yes	2031 (95.6)	93 (4.4)	1.92 (1.39–2.66,0.001)	1.41 (1.01–1.99, 0.044)
LVI	no	4686 (96.8)	155 (3.2)	-	-
	yes	29 (93.5)	2 (6.5)	2.08 (0.34-7.00, 0.318)	1.06 (0.16–3.85, 0.939)
CLNM	0	2980 (98.4)	47 (1.6)	-	-
	1–2	1142 (95.7)	51 (4.3)	2.83 (1.89–4.24,0.001)	2.91 (1.93–4.42,0.001)
	≥ 3	593 (91.0)	59 (9.0)	6.31 (4.26–9.38,0.001)	5.84 (3.83–8.93,0.001)

We proposed a scoring rule according to the results of the multivariate analysis: Tumor location: upper lobe - yes = 1, no = 0; Tumor size: >7 mm = 1, ≤ 7 mm = 0; ETE: yes = 1, no = 0; Number of CLNMs: ≥3 = 2, 1–2 = 1, 0 = 0. According to the above rules, the individual scores were added to obtain a total score. Overall, 1102 patients had a total score of zero, 1533 patients had a total score of 1, 1276 patients had a total score of 2, 692 patients had a total score of 3, 241 patients had a total score of 4, and 28 patients had a total score of 5. The rates of LLNM in patients with total scores of 0, 1, 2, 3, 4, and 5 were 0.2%, 1.8%, 2.8%, 8.5%, 11.2%, and 21.4%, respectively.

### Nomogram construction

**Fig. 1:**
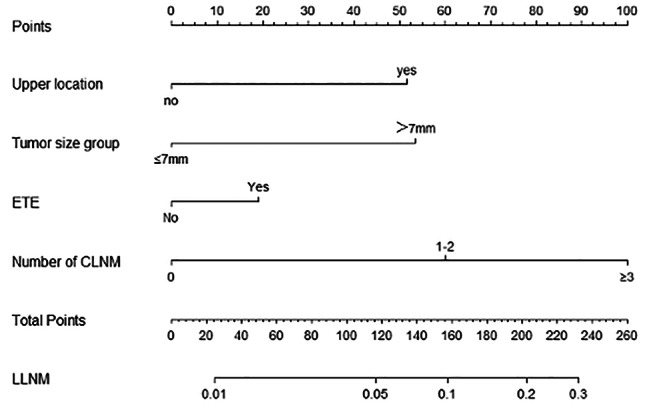
Nomogram for Predicting Lymph Node Metastasis in cN0 Unifocal Papillary Thyroid Microcarcinoma (PTMC) Patients

**Fig. 2:**
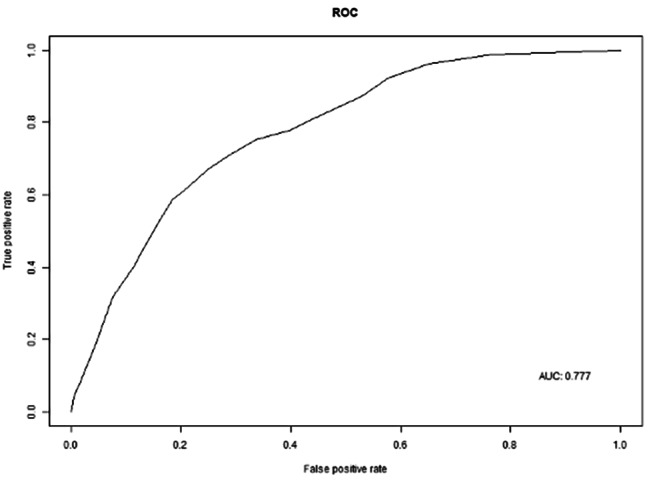
Receiver Operating Characteristic (ROC) Curve for Predicting Lymph Node Metastasis in cN0 Unifocal PTMC Patients

**Fig. 3:**
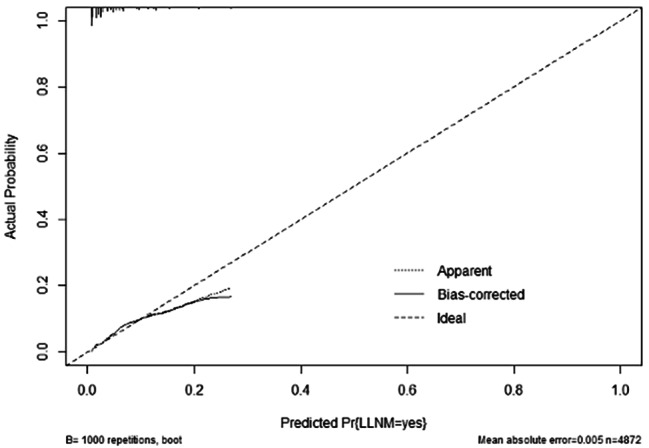
Calibration Curves of the Nomogram for Predicting Lymph Node Metastasis in cN0 Unifocal PTMC Patients

Based on the results of logistic regression analysis, a nomogram was constructed to predict LLNM in patients with cN0 unifocal PTMC, incorporating the four factors with non-zero coefficients: tumor location, tumor size, presence of ETE, and number of CLNMs (refer to Fig. 1). Notably, the number of CLNMs had the strongest contribution to the prediction model. The accuracy of the nomogram was verified using ROC curves, where the AUC was 0.777 with a 95% confidence interval (CI) ranging from 0.743 to 0.810 (refer to Fig. 2). The optimal cutoff score was identified as 89.6110, with a sensitivity of 70.7% and a specificity of 71.6%. When patients with cN0 unifocal PTMC obtains a total score > 89.6110, they have a significantly higher probability of LLNM. The calibration curve demonstrated excellent consistency between predicted and actual probabilities (refer to Fig. 3).

## Discussion

To the best of our knowledge, limited researches have been conducted on the risk factors for LLNM in cN0 PTMC patients, especially those with a large sample size from a single center [6, 10–12]. In this retrospective study, we analyzed data from 4872 patients with cN0 PTMC to investigate the risk factors for LLNM. The rate of occult LLNM was found to be 3.2%, which is consistent with previous studies (ranging from 3.7 to 7.5%) [6, 12]. Previous studies have recognized multifocality as a risk factor for LNM in PTMC [17, 18]. We analyzed the association between multifocality and the LLNM rate and found that patients with multifocal PTMC had a significantly higher LLNM rate (4.7%; 153/3249, p = 0.006). However, in multivariate analysis, multifocality was not found to be an independent risk factor (data not shown). Subsequently, we included only patients with unifocal PTMC to investigate the risk factors for LLNM.

Primary tumor size is a known prognostic factor for differentiated thyroid carcinoma [8], and previous studies reported a significant correlation between an increased rate of LNM and larger tumor size [19]. Yon et al. reviewed 490 patients with PTMC and found that tumor size was a significant factor associated with LLNM [20]. In our study, ROC curve analysis identified the meaningful tumor size cutoff value as 7.5 mm, and patients were accordingly divided into two groups (≤ 7 mm and > 7 mm). The rates of LLNM in these two groups were found to be 1.9% and 6.3%, respectively, with significant differences in both univariate and multivariate analyses, and tumor size > 7 mm was identified as an independent risk factor for LLNM. Zhang et al. [18] reviewed 1066 PTMC patients and found that tumor size > 6 mm was significantly associated with LLNM, while other studies reported that tumor size > 5 mm was an independent predictor of high prevalence of LLNM [17, 21]. The variation in cutoff values for tumor size in various studies may be attributed to differences in population demographics and sample sizes. Furthermore, the patients enrolled in our study all had unifocal tumors, which may have influenced the determination of the cutoff value.

Tumor location is widely recognized as a factor correlated with the incidence and severity of lymph node metastasis. The presence of the superior thyroid artery facilitates the flow of lymphatic fluid, thereby promoting the spread of tumor cells, which explains why tumors located in the upper thyroid lobe tend to readily metastasize to the lateral neck [22–24], and tumor location in the upper third of the thyroid lobe was found to be an independent risk factor for LLNM [22]. In our study, we also found that the incidence of LLNM was significantly higher in patients with tumors in the upper lobe compared to those in the middle and lower lobes (5.1% vs. 2.7%). Furthermore, multivariate analysis confirmed that tumor location in the upper lobe was an independent risk factor for LLNM, which is consistent with previous research findings [18, 21, 25].

Extrathyroidal extension (ETE) is recognized as one of the most crucial prognostic factors for PTC, and is a significant risk factor LNM [8, 10]. In the eighth edition staging system (2017), tumors with macro-ETE invading strap muscles or organs are restaged as T3b or T4, while tumors with micro-ETE are staged as T1/2 (≤ 4 cm) or T3a (> 4 cm) [16]. Studies have suggested that microscopic ETE is still a significant predictor of LLNM [17, 25]. In our study, we included both micro- and macro-ETE in our analysis. The incidence of LLNM was 4.4% among patients with ETE and 2.3% among those without ETE, with significant differences observed in both univariate and multivariate analyses. However, we did not find a significant difference between patients with macro-ETE (3.6%) and those with micro-ETE or intrathyroidal tumors (3.2%, p = 0.832). Similarly, Back K found no significant association between macro-ETE and LLNM [21]. This may be due to the small sample size of macro-ETE in patients with cN0 PTMC. Recent studies have revealed that thyroid capsule discontinuity and tumors located near the capsule by preoperative ultrasonography (US) examination are independent risk factors for LLNM in PTMC patients [25, 26]. Although we did not review the preoperative US characteristics of the tumors, we believe that tumors with ETE must be closely related to the thyroid capsule under US examination. Therefore, it is possible to estimate the probability of LLNM based on preoperative US examination, according to the relationship between the tumor and thyroid capsule, and also the tumor location as previously mentioned.

CLNM has been confirmed as an important risk factor for LLNM in previous studies [17, 20, 25, 27]. Lim et al. found that the average number of positive LNs in the central compartment was significantly associated with LLNM [20]. In the present study, we found that patients with 1–2 CLNMs (OR = 2.91, 95% CI 1.93–4.42; p < 0.001) or ≥ 3 CLNMs (OR = 5.84, 95% CI 3.83–8.93; p < 0.001) had a significantly higher risk of LLNM than patients with no CLNMs. Bohec et al. reported that patients with > 5 positive CLNMs had a higher risk of LLNM [27]. CLNM was also found to be a predicter for lateral neck recurrence in PTC patients [28], and a recent study in our center revealed that the number of CLNMs (> 3) was highly associated with lateral neck recurrence in patients with pN1a PTC [29]. Therefore, we believe that the number of CLNM is a valuable predictor of the probability of LLNM.

Tumor location in the upper lobe, tumor size > 7 mm, ETE, and CLNM, especially ≥ 3 positive LNs were identified as independent risk factors for LLNM of cN0 unifocal PTMC in the present cohort study. Based on these factors, we established a nomogram with an excellent predictive value (AUC = 0.777), and a score of ≥ 89.611 on the nomogram indicated high risk for LLNM. This nomogram can be useful in predicting the probability of LLNM and determining a personalized surgical approach for patients with PTMC, as well as guiding surgeons to carefully evaluate the lateral neck during follow-up.

There are limitations in our study, including the inherent features of a nonrandomized, retrospective cohort study. LND was only performed for those with enlarged LNs detected by preoperative ultrasound and diagnosed with positive LNs by frozen pathology, which may have led to missing cases with subclinical LLNM. The strength of the current study was that this study was conducted in one medical facility with a substantial sample size and strict inclusion criteria, leading to reliable results.

In conclusion, tumor location in the upper lobe, greater than 7 mm in size, ETE, and CLNMs, especially three or more, were independent risk factors for LLNM in cN0 unifocal PTMC. A nomogram can be utilized to estimate and predict the probability of LLNM. A prospective multicenter study is therefore necessary to minimize selection bias and verify our findings.

## Data Availability

The datasets used and/or analyzed during the current study available from the corresponding author on reasonable request.

## References

[1] Hedinger C, Williams ED, Sobin LH (1989). The WHO histological classification of thyroid tumors: a commentary on the second edition. Cancer.

[2] Lim H, Devesa SS, Sosa JA, Check D, Kitahara CM (2017). Trends in thyroid cancer incidence and mortality in the United States, 1974–2013. JAMA.

[3] Yu XM, Wan Y, Sippel RS, Chen H (2011). Should all papillary thyroid microcarcinomas be aggressively treated? An analysis of 18,445 cases. Ann Surg.

[4] Chow SM, Law SC, Chan JK, Au SK, Yau S, Lau WH (2003). Papillary microcarcinoma of the thyroid-prognostic significance of lymph node metastasis and multifocality. Cancer.

[5] Bramley MD, Harrison BJ (1996). Papillary microcarcinoma of the thyroid gland. Br J Surg.

[6] Wada N, Duh QY, Sugino K, Iwasaki H, Kameyama K, Mimura T, Ito K, Takami H, Takanashi Y (2003). Lymph node metastasis from 259 papillary thyroid microcarcinomas: frequency, pattern of occurrence and recurrence, and optimal strategy for neck dissection. Ann Surg.

[7] Gülben K, Berberoğlu U, Celen O, Mersin HH (2008). Incidental papillary microcarcinoma of the thyroid–factors affecting lymph node metastasis. Langenbecks Arch Surg.

[8] Haugen BR, Alexander EK, Bible KC, Doherty GM, Mandel SJ, Nikiforov YE, Pacini F, Randolph GW, Sawka AM, Schlumberger M, Schuff KG, Sherman SI, Sosa JA, Steward DL, Tuttle RM, Wartofsky L (2016). 2015 american thyroid Association Management Guidelines for adult patients with thyroid nodules and differentiated thyroid Cancer: the american thyroid Association Guidelines Task Force on thyroid nodules and differentiated thyroid Cancer. Thyroid.

[9] Wen X, Jin Q, Cen X, Qiu M, Wu Z (2022). Clinicopathologic predictors of central lymph node metastases in clinical node-negative papillary thyroid microcarcinoma: a systematic review and meta-analysis. World J Surg Oncol.

[10] Chung YS, Kim JY, Bae JS, Song BJ, Kim JS, Jeon HM, Jeong SS, Kim EK, Park WC (2009). Lateral lymph node metastasis in papillary thyroid carcinoma: results of therapeutic lymph node dissection. Thyroid.

[11] So YK, Son YI, Hong SD, Seo MY, Baek CH, Jeong HS, Chung MK (2010). Subclinical lymph node metastasis in papillary thyroid microcarcinoma: a study of 551 resections. Surgery.

[12] Parvathareddy SK, Siraj AK, Annaiyappanaidu P, Siraj N, Al-Sobhi SS, Al-Dayel F, Al-Kuraya KS (2022). Risk factors for cervical lymph Node Metastasis in Middle Eastern Papillary thyroid Microcarcinoma. J Clin Med.

[13] Pisanu A, Reccia I, Nardello O, Uccheddu A (2009). Risk factors for nodal metastasis and recurrence among patients with papillary thyroid microcarcinoma: differences in clinical relevance between nonincidental and incidental tumors. World J Surg.

[14] Siddiqui S, White MG, Antic T, Grogan RH, Angelos P, Kaplan EL, Cipriani NA (2016). Clinical and pathologic predictors of Lymph Node Metastasis and recurrence in papillary thyroid microcarcinoma. Thyroid.

[15] Kim E, Park JS, Son KR, Kim JH, Jeon SJ, Na DG (2008). Preoperative diagnosis of cervical metastatic lymph nodes in papillary thyroid carcinoma: comparison of ultrasound, computed tomography, and combined ultrasound with computed tomography. Thyroid.

[16] Amin MB, Edge SB, Greene FL (2017). AJCC cancer staging manual.

[17] Kim SK, Park I, Woo JW, Lee JH, Choe JH, Kim JH, Kim JS (2016). Predictive factors for Lymph Node Metastasis in Papillary thyroid Microcarcinoma. Ann Surg Oncol.

[18] Zhang L, Wei WJ, Ji QH, Zhu YX, Wang ZY, Wang Y, Huang CP, Shen Q, Li DS, Wu Y (2012). Risk factors for neck nodal metastasis in papillary thyroid microcarcinoma: a study of 1066 patients. J Clin Endocrinol Metab.

[19] Das R, Rahman T, Das AK, Das K, Das A, Kakati K, Das R (2022). Pattern of nodal metastasis in relation to size of the primary Tumour in Well-Differentiated thyroid carcinoma. Indian J Otolaryngol Head Neck Surg.

[20] Lim YS, Lee JC, Lee YS, Lee BJ, Wang SG, Son SM, Kim IJ (2011). Lateral cervical lymph node metastases from papillary thyroid carcinoma: predictive factors of nodal metastasis. Surgery.

[21] Back K, Kim JS, Kim JH, Choe JH (2019). Superior located papillary thyroid Microcarcinoma is a risk factor for lateral lymph node metastasis. Ann Surg Oncol.

[22] Ducoudray R, Trésallet C, Godiris-Petit G, Tissier F, Leenhardt L, Menegaux F (2013). Prophylactic lymph node dissection in papillary thyroid carcinoma: is there a place for lateral neck dissection?. World J Surg.

[23] Lee YS, Shin SC, Lim YS, Lee JC, Wang SG, Son SM, Kim IJ, Lee BJ (2014). Tumor location-dependent skip lateral cervical lymph node metastasis in papillary thyroid cancer. Head Neck.

[24] Ito Y, Tomoda C, Uruno T, Takamura Y, Miya A, Kobayashi K, Matsuzuka F, Kuma K, Miyauchi A (2004). Papillary microcarcinoma of the thyroid: how should it be treated?. World J Surg.

[25] Kwak JY, Kim EK, Kim MJ, Son EJ, Chung WY, Park CS, Nam KH (2009). Papillary microcarcinoma of the thyroid: predicting factors of lateral neck node metastasis. Ann Surg Oncol.

[26] Wang D, Zhu J, Deng C, Yang Z, Hu D, Shu X, Yu P, Su X (2022). Preoperative and pathological predictive factors of central lymph node metastasis in papillary thyroid microcarcinoma. Auris Nasus Larynx.

[27] Bohec H, Breuskin I, Hadoux J, Schlumberger M, Leboulleux S, Hartl DM (2019). Occult Contralateral lateral lymph node metastases in unilateral N1b papillary thyroid carcinoma. World J Surg.

[28] Giordano D, Frasoldati A, Kasperbauer JL, Gabrielli E, Pernice C, Zini M, Pedroni C, Cavuto S, Barbieri V (2015). Lateral neck recurrence from papillary thyroid carcinoma: predictive factors and prognostic significance. Laryngoscope.

[29] Xu S, Huang H, Huang Y, Wang X, Xu Z, Liu S, Liu J (2022). Risk stratification of lateral neck recurrence for patients with pN1a papillary thyroid cancer. BMC Cancer.

